# Leveraging TIS-Enhanced Crayfish Optimization Algorithm for High-Precision Prediction of Long-Term Achievement in Mathematical Elite Talents

**DOI:** 10.3390/biomimetics11030194

**Published:** 2026-03-06

**Authors:** Shenrun Pan, Qinghua Chen

**Affiliations:** School of Mathematics and Statistics, Fujian Normal University, Fuzhou 350117, China; panshenrun@163.com

**Keywords:** mathematical giftedness, Professional Achievement in Mathematics (PAM), early identification, emotion regulation, model robustness

## Abstract

Traditional talent identification systems often rely on static assessments and overlook the dynamic nature of long-term development. To address this limitation, this study proposes a biomimetic predictive framework inspired by crayfish behavioral ecology. The Crayfish Optimization Algorithm (COA), derived from adaptive foraging and competition mechanisms observed in crayfish, is enhanced through a Thinking Innovation Strategy (TIS) to form TISCOA for hyperparameter optimization of a Gradient Boosting Decision Tree model. Using a five-year longitudinal dataset of 160 elite mathematical students, the framework models Professional Achievement in Mathematics (PAM) from multidimensional baseline indicators. Comparative experiments with multiple metaheuristic optimizers show that the proposed approach achieves stable generalization performance within the examined cohort. Feature attribution analysis indicates that non-cognitive factors, particularly Emotion Regulation, contribute substantially to long-term outcomes, while temporal variables such as the Latency Period further shape developmental trajectories. Residual analysis highlights heterogeneous patterns that may reflect unobserved contextual influences. Overall, the study demonstrates how a biologically inspired optimization mechanism can support interpretable and stability-oriented longitudinal prediction in small-sample educational settings.

## 1. Introduction

The development of high-level mathematical talent is a long-term and dynamic process shaped by the interaction between cognitive potential and environmental and psychological catalysts [1]. Although early identification remains central to talent cultivation, empirical evidence indicates that initial cognitive advantage does not guarantee sustained professional achievement. The Expert Performance framework emphasizes that excellence in complex domains emerges from prolonged developmental trajectories rather than static indicators of intelligence [2]. Mathematical giftedness often unfolds through nonlinear growth and developmental variability that cannot be captured by cross-sectional assessments. Longitudinal and trend-based modeling is therefore essential for bridging the gap between early promise and realized expertise.

Traditional identification systems rely heavily on standardized intelligence and mathematics achievement tests, including nationally administered cognitive ability assessments and curriculum-based examinations. While such instruments provide structured benchmarks of reasoning and academic proficiency, they do not account for developmental dynamics or non-cognitive moderators. Contemporary talent development theories, particularly the Differentiated Model of Giftedness and Talent, conceptualize excellence as a dynamic transformation process in which natural abilities evolve into professional competence through intrapersonal and environmental catalysts [3]. In this study, long-term outcome is operationalized as Professional Achievement in Mathematics, a composite index integrating academic performance, competitive achievement, and research potential as detailed in Section 2.1.1. Preliminary analysis reveals a near-zero correlation between baseline performance and five-year achievement, highlighting the decoupling between early status and long-term outcome.

Recent advances in Educational Data Mining and Learning Analytics demonstrate the effectiveness of machine learning methods for modeling academic progression, dropout risk, and longitudinal performance trajectories [4,5,6]. Tree-based ensemble models and cross-validation strategies are widely used in structured educational datasets due to their robustness and interpretability [7]. Longitudinal predictive modeling increasingly emphasizes stability-oriented evaluation rather than single-point accuracy, particularly in moderate-sized cohorts [8]. At the same time, psychometric research confirms the predictive relevance of non-cognitive constructs such as emotional regulation, persistence, and academic resilience for long-term academic outcomes [9,10]. Despite these developments, applications of machine learning to multi-year giftedness trajectories, especially in mathematics, remain comparatively limited. Few studies integrate cognitive, non-cognitive, and contextual variables within a stability-focused longitudinal framework.

Methodologically, longitudinal talent datasets are typically small, high dimensional, nonlinear, and heterogeneous. Conventional statistical approaches often struggle with such complexity. Tree-based ensemble models such as Gradient Boosting Decision Trees perform well on structured tabular data and are relatively robust in small-sample settings. However, their performance depends strongly on hyperparameter configuration, and optimization in non-convex search spaces is challenging. Although metaheuristic algorithms are commonly used for tuning, many studies prioritize marginal reductions in absolute error while giving less attention to generalization stability.

To address these challenges, this study introduces a biomimetic optimization mechanism based on the Crayfish Optimization Algorithm, which is inspired by adaptive foraging and competitive behaviors observed in crayfish populations. By incorporating a Thinking Innovation Strategy, we develop a TIS-enhanced COA tailored to small-scale longitudinal talent prediction. The proposed framework emphasizes stability-oriented search dynamics aligned with the longitudinal modeling objective.

The main contributions of this work are summarized as follows:

1. A longitudinal stability oriented predictive framework.

We construct an integrated modeling pipeline that explicitly targets five-year professional achievement using baseline multidimensional indicators, shifting the focus from static classification to trend-based prediction.

2. TISCOA enhanced hyperparameter optimization strategy.

By incorporating a Thinking Innovation Strategy into the Crayfish Optimization Algorithm, we develop a stability driven optimization mechanism for tuning GBDT hyperparameters into non-convex search spaces. The framework demonstrates statistically significant improvement in mean squared error, emphasizing generalization robustness over marginal accuracy gains.

3. A dual layer interpretability architecture.

We combine internal feature gain analysis with SHAP-based attribution to distinguish structural gatekeepers from marginal fluctuation drivers, providing transparent insight into the mechanisms underlying long term mathematical achievement.

4. A diagnostic residual analysis mechanism.

Beyond predictive performance, we implement systematic residual examinations to identify developmental deviations such as under prediction and over prediction cases, extending the model from a forecasting tool to a potential intervention support instrument.

Collectively, these contributions establish a robust and interpretable framework for modeling longitudinal mathematical talent development while prioritizing generalization stability in small-scale educational datasets.

## 2. Materials and Methods

### 2.1. Participants and Data Collection

#### 2.1.1. Operational Definition of PAM

Professional Achievement in Mathematics (PAM) was measured at Year 5 as a composite index ranging from 0 to 10. The index integrates three components: Academic Performance (40%), Competitive Achievement (30%), and Research Potential (30%).

Academic Performance reflects weighted grades from advanced mathematics curricula. Competitive Achievement is based on verified rankings in national or international mathematics competitions. Research Potential was evaluated using standardized expert rubric assessing conceptual depth and analytical reasoning.

All components were normalized before aggregation. The weighting scheme was determined through expert consensus among mathematics educators and talent development specialists. Qualitative assessments were independently cross validated by an expert panel to ensure validity and scoring consistency. The resulting PAM distribution approximated normality, supporting subsequent regression and residual analyses.

#### 2.1.2. Participant Selection and Cohort Characteristics

The cohort comprises 160 students enrolled in a structured mathematical talent development program and tracked continuously for five years. Identification at Year 0 followed a multistage screening procedure including documented high mathematical aptitude, teacher nomination, competitive mathematics performance, and standardized assessment benchmarks. Inclusion required sustained engagement in advanced mathematics curricula.

All predictors used in this study were measured at baseline (Year 0) or derived from baseline assessments. No post-outcome variables were included in the predictive design.

Although modest in size relative to large-scale educational datasets, the sample represents a highly selective elite population within a specialized training pipeline. Ensemble tree-based models such as GBDT have demonstrated strong performance in structured moderate-sized tabular datasets [11], and the nested validation protocol further mitigates overfitting risk.

#### 2.1.3. Multi-Dimensional Feature Architecture

Seventeen primary predictors were defined at baseline and grouped into four domains: (a) Baseline traits, including demographic characteristics and initial performance indicators; (b) Cognitive indicators, including Essence Grasping and Acceptance of New Definitions; (c) Intrapersonal characteristics, including Emotion Regulation and persistence; and (d) Environmental exposure variables, including structured advanced training intensity and Psychological Support.

Psychological and non-cognitive indicators were collected using structured Likert-type self-report instruments administered within the program. Internal consistency was evaluated prior to modeling, and composite scores were standardized before inclusion.

Environmental and Psychological Support variables represent structured program-level exposures recorded prior to Year 5 outcome measurement. These variables were available at baseline or as predefined baseline-aggregated summaries and are treated as contextual predictors rather than causal treatment effects.

All predictors used for model training were strictly baseline or baseline-aggregated measures.

#### 2.1.4. Data Preprocessing and Interaction Terms

All numerical predictors were standardized prior to modeling. Scaling parameters were estimated exclusively from the training set and subsequently applied to the independent test set to prevent information leakage.

To capture potential synergy effects between intrapersonal traits and environmental exposures, interaction terms were constructed based on theoretical relevance [12,13]. These interaction terms were predefined and incorporated within the 17-feature design matrix; they did not expand the dimensionality beyond the specified predictor set.

Missing values were minimal and handled exclusively within training folds.

### 2.2. Longitudinal Predictive Pipeline

**Figure 1:**
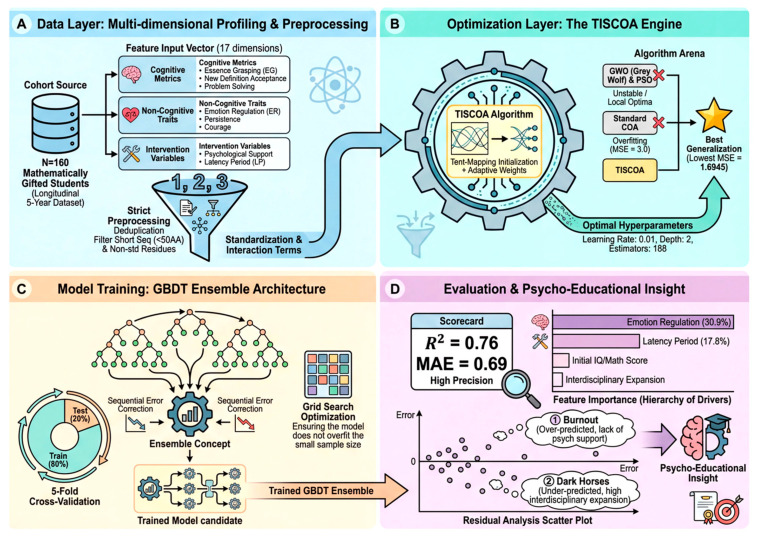
Conceptual framework of the GBDT-based longitudinal predictive model.

The proposed framework is organized as a longitudinal analytical pipeline that maps baseline multidimensional features to five-year professional achievement, as illustrated in Figure 1.

The dataset was partitioned into an 80% training set and a 20% independent test set using stratified random splitting. Hyperparameter optimization was conducted exclusively within the training set using an inner fivefold cross-validation loop. The independent test set remained completely unseen during optimization, eliminating optimizer-induced information leakage.

Headline performance metrics (R^2^, MAE, MSE) reported in Section 3 were computed exclusively on the independent test set. Cross-validation scores were used solely for hyperparameter selection and not for final performance reporting.

### 2.3. Predictive Modeling

The GBDT algorithm was selected due to its ability to model nonlinear interactions and heterogeneous feature types in structured tabular data [14]. Compared with high-capacity neural networks, GBDT is less prone to overfitting in moderate-sized samples.

Hyperparameter tuning was performed using the nested validation framework described above. The final configuration included a learning rate of 0.1, 100 estimators, and a maximum tree depth of 3.

Model interpretability was assessed using cumulative reduction in impurity across trees, enabling identification of dominant predictors.

To evaluate reproducibility and optimizer stability, each metaheuristic algorithm was executed for 30 independent runs with different random seeds. For each run, optimized hyperparameters were applied within the same nested validation framework. Final performance statistics are reported as mean ± standard deviation based on independent test-set results.

### 2.4. Optimization Process of Crayfish Optimization Algorithm

Crayfish Optimization Algorithm (COA) is a metaheuristic algorithm proposed by Jia et al. in 2023 [15]. Its complete optimization process consists of the following phases: population initialization, temperature and crayfish intake definition, summer resort behavior, competition, and foraging.

#### 2.4.1. Initialize Population

In a multi-dimensional optimization problem, let *N* and *d* represent the population size and dimensionality, respectively. Each crayfish corresponds to a 1 × *d* matrix, where each column denotes a potential solution to the problem. The initialization of COA involves randomly generating *N* candidate solutions within the specified lower and upper bounds. This process is expressed as [15]:

(1) $$X={\left[X_{1},X_{2},\ldots ,X_{N}\right]}^{T}=\left[\begin{array}{cc} \begin{array}{cc} X_{11} & X_{12}\\ X_{21} & X_{22} \end{array} & \begin{array}{cc} \ldots & X_{1d}\\ \ldots & X_{2d} \end{array}\\ \begin{array}{cc} \vdots & \vdots \\ X_{N1} & X_{N2} \end{array} & \begin{array}{cc} \ddots & \vdots \\ \ldots & X_{Nd} \end{array} \end{array}\right]$$

(2) $$X_{i,\;j}={lb}_{j}+({ub}_{j}-{lb}_{j})\cdot rand$$

where *X* denotes the position of the population, ${lb}_{j}$ and ${ub}_{j}$ represent the lower and upper bounds of the *j*-th dimension, respectively, and rand is a random number uniformly distributed within the range [0, 1].

#### 2.4.2. Define Temperature and Intake of Crayfish

The change in ambient temperature influences the behavior of crayfish by acting as a stochastic switch between exploration and exploitation. Specifically, temperature *T* determines whether the population engages in high-temperature avoidance behaviors (Summer Resort and Competition) or low-temperature resource acquisition (Foraging), thereby maintaining search diversity and convergence speed. The environmental temperature, denoted as *T*, is defined as follows [15]:

(3) T=rand·15+20.

The food intake of crayfish approximately follows a normal distribution. The corresponding mathematical model and the intake amount *P* under different temperatures are expressed as follows [16]:

(4) $$P=C_{1}\cdot (\frac{1}{\sqrt{2\;\times \;\pi }\;\times \;\sigma }\cdot exp(-\frac{{\left(T\;-\;\mu \right)}^{2}}{2\sigma^{2}}))$$

where *μ* denotes the most favorable temperature for crayfish, and *σ* and $C_{1}$ are parameters used to control the crayfish’s food intake under varying temperatures.

#### 2.4.3. Summer Resort

When the ambient temperature exceeds 30 °C (*T* > 30), crayfish will retreat into their burrows to avoid the heat. The cave position, $X_{shade}$, is defined as follows [15]:

(5) $$X_{shade}\;=\;\frac{X_{G}\;+{\;X}_{L}}{2}$$

where $X_{G}$ represents the optimal position obtained in the current iteration, and $X_{L}$ denotes the best position acquired from the previous generation after population update.

The competition among crayfish for caves is regarded as a stochastic event. During the contention process, there is a 50% probability (rand < 0.5) that no other crayfish will compete for the target cave. In this scenario, the crayfish directly enters the cave to avoid the heat. This process is expressed as follows [15]:

(6) $$X_{i}(t+1)=X_{i}(t)+C_{2}\cdot rand\cdot (X_{shade}-X_{i}(t))$$

where *t* represents the current iteration number, and $C_{2}=2-t/T_{max}$ denotes the decreasing curve that is affected by the maximum number of iterations $T_{max}$.

#### 2.4.4. Competition

When the ambient temperature exceeds 30 °C (*T* > 30) and rand ≥ 0.5, it indicates that other crayfish have also selected the target burrow. In this case, the crayfish will compete with others for the burrow. This process is expressed as follows [15]:

(7) $$X_{i,\;j}(t+1)=X_{i,\;j}(t)-X_{z,\;j}(t)+{\left(X_{shade}\right)}_{j}$$

(8) z=round(rand·(N−1)+1)

where *z* represents the position index of a randomly selected individual crayfish, and round (·) function is used to round a number to the nearest integer.

#### 2.4.5. Foraging

When the ambient temperature is below 30 °C (*T* ≤ 30), crayfish begin foraging. The food position $X_{food}$ is defined as follows [8]:

(9) $$X_{food}=X_{G}.$$

During feeding, crayfish decide whether to shred the food based on its size. If the food is appropriately sized, they consume it directly. The food size *Q* is defined as follows [16]:

(10) $$Q=C_{3}\cdot rand\cdot \frac{{fitness}_{i}}{{fitness}_{food}}$$

where $C_{3}$ denotes the food factor, with a value of 3. ${fitness}_{i}$ and ${fitness}_{food}$ represent the fitness value of the *i*-th crayfish and the fitness value at the food position, respectively. If $Q>(C_{3}+1)/2$, it indicates that the food is too large. In this case, the crayfish will shred the food. This process is expressed as follows [15]:

(11) $$X_{food}=exp(-\frac{1}{Q})\cdot X_{food}.$$

After the food is shredded, the crayfish will alternately grasp and ingest the food using its second and third pairs of walking legs. This process is expressed as follows [15]:

(12) $$X_{i,\;j}(t+1)=X_{i,\;j}(t)+{\left(X_{food}\right)}_{j}\cdot P\cdot (cos(2\cdot \pi \cdot rand)-sin(2\cdot \pi \cdot rand)).$$

If $Q\leq (C_{3}+1)/2$, the crayfish will directly move toward the food and begin feeding. This process is expressed as follows [15]:

(13) $$X_{i}(t+1)=(X_{i}(t)-X_{food})\times P+P\cdot rand\cdot X_{i}(t).$$

This study fully leveraged the advantages of COA, namely its efficient optimization capabilities and global search ability, to precisely solve high-precision prediction problems, thereby providing a reliable screening solution for the cultivation of mathematical talents.

### 2.5. TISCOA Algorithm

#### 2.5.1. Conceptual Advantages over Baseline Optimizers

The Thinking Innovation Strategy (TIS) was proposed as a plug-and-play enhancement for metaheuristic algorithms [16]. It improves exploration and exploitation balance by incorporating Information Events (IE), Depth of Knowledge (DOK), and Imagination (IM) mechanisms [17].

By integrating TIS into COA, the improved optimizer (TISCOA) enhances population diversity and reduces stagnation risk in non-convex search spaces.

#### 2.5.2. Mathematical Modeling of TIS Mechanisms

Each candidate solution *Xi* corresponds to a GBDT hyperparameter vector [*n*, *lr*, *d*]. The fitness function was defined as the mean MSE across fivefold cross-validation, prioritizing generalization stability. MAE was recorded for supplementary comparison.

All optimizers were executed under identical computational budgets to ensure fairness of comparison.

Final optimized hyperparameters were used to train the GBDT model on the full training set, and R^2^, MAE, and MSE were computed on the independent test set.

#### 2.5.3. TISCOA-GBDT Coupling for Hyperparameter Optimization

Due to the reasons mentioned above, TIS is employed in this study to enhance the performance of the COA algorithm. The improved COA is named TISCOA. The principles of TIS are embedded in this algorithm and its key role are as follows:

**Information events:** The *IE* is an important basis for identifying individual crayfish, and it can provide crucial information for the exploration and development of algorithms. In the initial stage of TISCOA, a crayfish individual is randomly selected and stored in the *IE*. As the algorithm progresses, the survival of the fittest approach is adopted to retain outstanding crayfish individuals, preparing for the subsequent innovative thinking process.

**Innovative thinking:** The TIS first endows individual crayfish with the ability to think innovatively. When confronted with complex problems, individual crayfish often rely on their own *DOK* to seek solutions, and the *DOK* is represented as:

(14) DOK=DOK1+DOK2

(15) $$DOK1=\sqrt{\frac{t}{T_{max}}}+C$$

(16) $$DOK2=t^{10}+C$$

where *DOK*1 and *DOK*2 respectively denote the accumulated knowledge and experience over time and the continuous acquisition and storage of information, and *C* denotes the constant with a value of 0.5.

Secondly, based on the individual cognition of crayfish, during this process, TIS also endows the individual crayfish with the ability to imagine, and the *IM* is represented as:

(17) IM=π·IE·rand.

Finally, to enable the crayfish individuals to generate novel and valuable outcomes, TIS innovates by integrating or transforming their *DOK* and *IM*, and guides the crayfish individuals to think in multiple directions. This process is represented as:

(18) $$X_{new}=\tan\left(IM-0.5\cdot \pi )+(\frac{X}{DOK}+IE\right)$$

where $X_{new}$ represents the updated position of the population after the action of TIS.

In this study, the TISCOA algorithm is employed to globally optimize the key hyperparameters of the GBDT model to achieve more robust five-year PAM prediction performance. Specifically, we construct the three core hyperparameters of GBDT—learning rate (Learning Rate), the number of weak learners (Estimators), and maximum tree depth (Max Depth)—as a continuous/discrete hybrid decision vector to be optimized and iteratively searched within a 5-fold cross-validation framework. This setting is consistent with the subsequent comparative experiments: all meta-heuristic optimizers are tasked with “tuning GBDT hyperparameters within 5-fold cross-validation” to ensure fair comparability among different algorithms. To ensure fair comparability among different algorithms, all meta-heuristic optimizers were tasked with tuning GBDT hyperparameters within the same computational budget. Specifically, a uniform population size of 30 and a maximum of 100 iterations were applied to each optimizer, totaling 3000 function evaluations per run. This ensures that any observed performance gains are strictly attributable to the algorithmic mechanisms rather than variations in search effort.

To enable TISCOA to directly serve the GBDT hyperparameter tuning task, this study adopts the following “candidate solution encoding—fitness evaluation—innovation update” coupled strategy:

**Candidate solution encoding:** Each position vector *X_i_* of a crayfish individual corresponds to a set of GBDT hyperparameter combinations, denoted as *X_i_* = [*n_i_*, *lr_i_*, *d_i_*], where *n_i_* represents the number of trees, *lr_i_* represents the learning rate, and *d_i_* represents the maximum depth. Since *n_i_* and *d_i_* are integer variables, after iterative updates, they are mapped back to the feasible domain through rounding/truncation operations; *lr_i_*, as a continuous variable, is directly retained.

**Fitness function definition:** Considering that the longitudinal prediction task places more emphasis on “extreme error risk” and model stability, this study takes the mean squared error (MSE) as the main optimization objective, that is, the average MSE under 5-fold cross-validation is used as the fitness value to be minimized; meanwhile, the MAE is recorded for auxiliary comparison and interpretation, but it is not the main optimization objective. This setting is consistent with the modeling stance emphasized in this paper that “stability takes priority over marginal accuracy improvement”.

**Innovative update driven by TIS:** In each iteration, the algorithm first retains representative excellent individuals through *IE*, then characterizes the “experience accumulation—information absorption—leap exploration” ability of individuals based on *DOK* and *IM* and realizes the position update under multi-directional thinking through Equation (18). This update mechanism can enhance global exploration in the early stage and strengthen local development in the middle and later stages, thereby reducing the risk of COA falling into local optimal or overfitting hyperparameter combinations when tuning parameters with complex, highly heterogeneous small sample data.

Ultimately, when the maximum number of evaluations is reached, TISCOA outputs the global optimal individual X*, and uses the corresponding (n*, lr*, d*) as the final hyperparameter configuration for GBDT to train the complete model and report metrics such as R^2^, MAE, and MSE on an independent test set. Through this end-to-end coupled process of “TISCOA-GBDT”, this paper achieves more reliable long-term predictions of longitudinal talent development trajectories while ensuring generalization stability.

#### 2.5.4. Model Interpretability and Feature Prioritization

To ensure a robust and transparent interpretation of the talent development drivers, this study employs a dual-layered interpretability framework. This approach is designed to move beyond “black-box” predictions and establish a granular hierarchy of influence.

First, we calculate the internal feature importance of the GBDT model based on the total reduction in information gain (Gini impurity) associated with each feature split. This metric captures the cumulative utility of a variable across the entire forest, identifying “structural gatekeepers” such as Emotion Regulation (30.9%)—that are essential for defining the model’s decision logic and overall trajectory.

Second, to mitigate potential measurement bias and verify the stability of our conclusions, we integrate SHAP (Shapley Additive Explanations) values. Unlike default tree-based metrics, SHAP provides a consistent attribution of importance by considering the marginal contribution of each feature across all possible feature combinations.

By synthesizing these dual perspectives, we can distinguish between variables that act as foundational catalysts (e.g., non-cognitive traits that stabilize the development process) and those that serve as direct drivers of score fluctuations (e.g., specific cognitive indicators like New Definition Acceptance). This cross-verification ensures that the identified hierarchy of drivers is not an artifact of feature variance but reflects the multi-layered nature of elite mathematical development.

## 3. Numerical Results

### 3.1. Correlation Matrix

**Figure 2:**
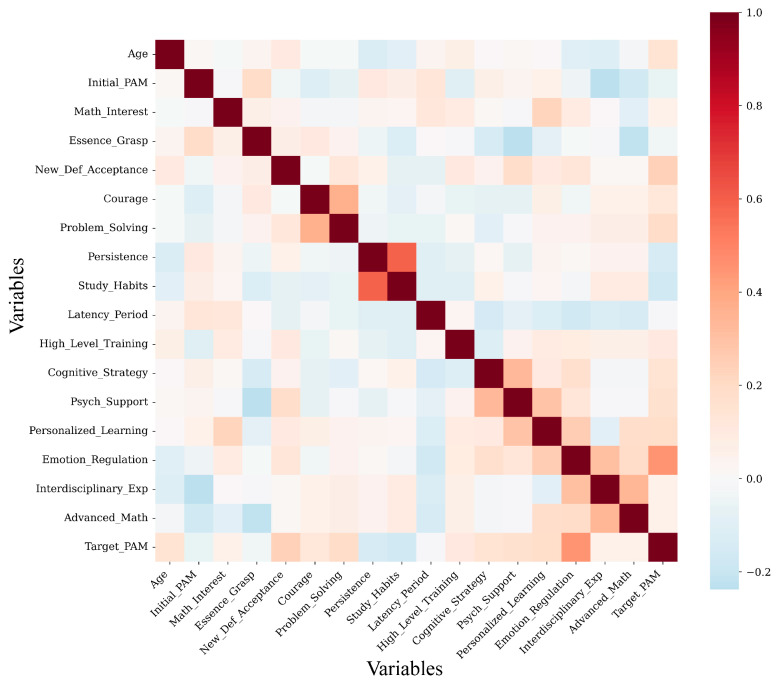
Inter-correlation matrix of 18 variables including 17 predictive indicators and the target PAM outcome.

Pearson correlation analysis (Figure 2) reveals a clear divergence between early status indicators and long-term developmental outcomes. Among all examined variables, Emotion Regulation exhibits the strongest positive linear association with five-year professional achievement (*r* = 0.45), indicating that individuals with greater emotional self-regulation capacity tend to demonstrate more stable and sustained growth over extended developmental periods. This finding supports theoretical models that emphasize the role of intrapersonal catalysts in transforming early aptitude into realized expertise.

In contrast, Initial PAM scores display a negligible and slightly negative correlation with long-term achievement (*r* = −0.06), suggesting a pronounced “decoupling” between early performance status and subsequent professional development. This counterintuitive result implies that early academic excellence does not necessarily translate into sustained advancement and may, in some cases, mask latent vulnerabilities such as motivational decline or maladaptive stress responses. Collectively, these results underscore the limitations of snapshot-based assessments and highlight the necessity of incorporating dynamic psychological indicators when modeling longitudinal talent trajectories.

### 3.2. Predictive Performance and Feature Importance

**Figure 3:**
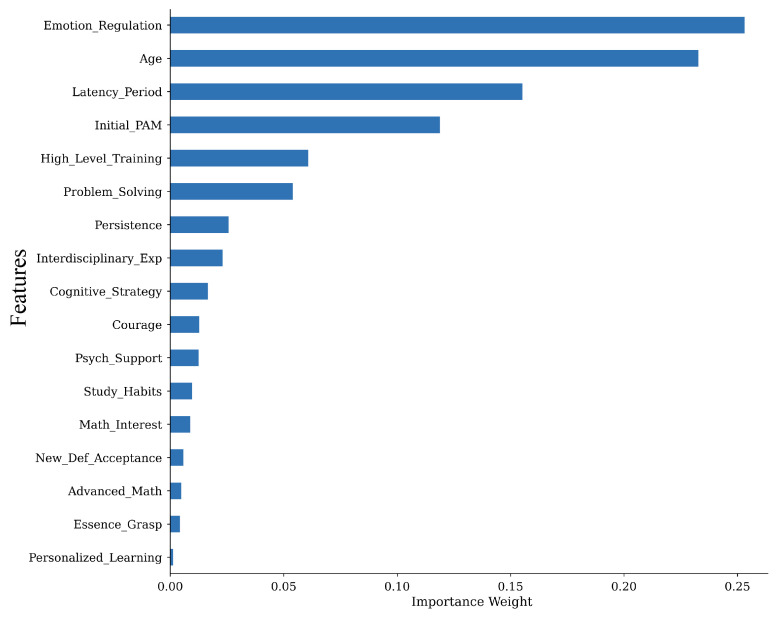
GBDT feature importance.

In terms of model evaluation and factor prioritization, the GBDT framework demonstrated stable predictive performance within the examined cohort, yielding an R^2^ coefficient of 0.7647 on the independent test set. This indicates that the integrated model, accounting for cognitive, non-cognitive, and intervention-based variables, can explain approximately 76.5% of the total variance in five-year mathematical achievement. Furthermore, the MAE was recorded at 0.6976, suggesting that the model’s predictions typically deviate by less than 0.7 units on the PAM scale. These results indicate substantially explained variance within the examined cohort. Beyond global performance metrics, the feature importance analysis provides a granular look at the hierarchy of talent drivers, revealing that non-cognitive factors exercise a dominant influence over the predictive gain [18]. As illustrated in Figure 3, Emotion Regulation emerged as the most decisive predictor, accounting for 30.9% of the model’s total relative importance. This highlights the strong predictive association between emotion regulation and long-term mathematical achievement. Additionally, the Latency Period—representing the temporal gap between initial identification and systematic testing—contributed 17.8% to the predictive power. The significant weight of this temporal variable suggests that the timing and “trend” of a student’s development are more indicative of their future potential than static, cross-sectional ability scores alone. By shifting the focus from purely cognitive aptitude to these dynamic behavioral and temporal markers, the model offers a more nuanced evidence-based approach to talent identification.

**Figure 4:**
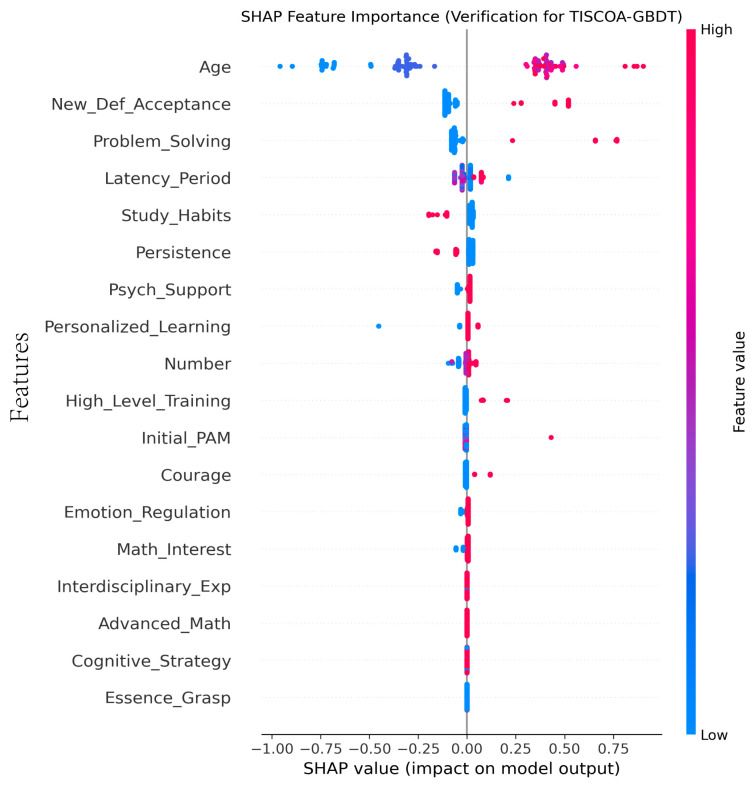
Shapley additive explanations analysis.

To validate the robustness of the feature importance derived from the GBDT model, we supplementary conducted a SHAP analysis (Figure 4). This model-agnostic approach ensures that the dominance of non-cognitive factors is not an artifact of feature variance. The SHAP results consistently identify Emotion Regulation as the primary driver of Professional Achievement in Mathematics (PAM), corroborating our initial findings that psychological stability acts as a critical catalyst in long-term talent development.

### 3.3. Residual Analysis and Outlier Detection

**Figure 5:**
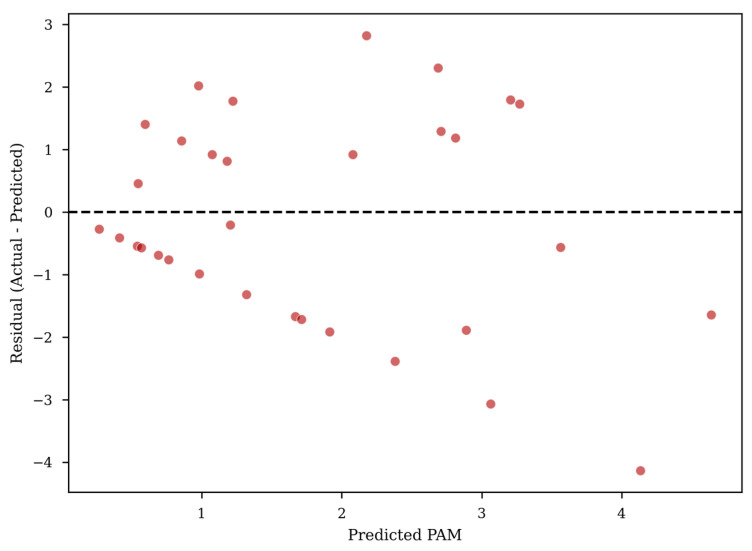
Residual analysis.

To assess the robustness of the predictive framework and identify potential sources of variance, a residual analysis was performed on the test set results. As illustrated in Figure 5, the majority of the model’s predictions are tightly clustered around the zero-error baseline, with most residuals falling within a narrow margin of ±0.5 points on the PAM scale. Visual inspection suggests no apparent heteroscedastic trend across achievement levels. However, this observation is descriptive rather than inferential [19]. Given the 0–10 PAM scale, an absolute error of 1.0 corresponds to 10% of the full-scale range. This threshold is used descriptively to identify relatively large deviations rather than as a formal statistical cutoff. These outliers primarily fall into two categories: “Dark Horses” (Under-prediction) and “Over-prediction” cases. Students categorized as “Dark Horses” those whose actual achievement significantly exceeded model expectations—were found to possess high scores in Interdisciplinary Expansion. This suggests that exposure to diverse academic fields may act as a non-linear “catalyst,” which may be associated with later-stage performance improvements. Conversely, the “Over-prediction” cases involved students who possessed superior cognitive traits and high initial potential scores but ultimately failed to reach their predicted PAM levels. A qualitative review of these instances revealed a recurring lack of Psychological Support. This finding emphasizes that high cognitive aptitude is not self-sustaining; without targeted emotional and environmental scaffolding, which may reflect heterogeneous developmental trajectories or psychosocial strain [20]. By identifying these specific deviations, the residual analysis transitions the study from a general prediction to a focused “risk-management” tool, indicating cases where additional human-led review may be warranted.

### 3.4. Comparative Performance of Optimization Algorithms

**Figure 6:**
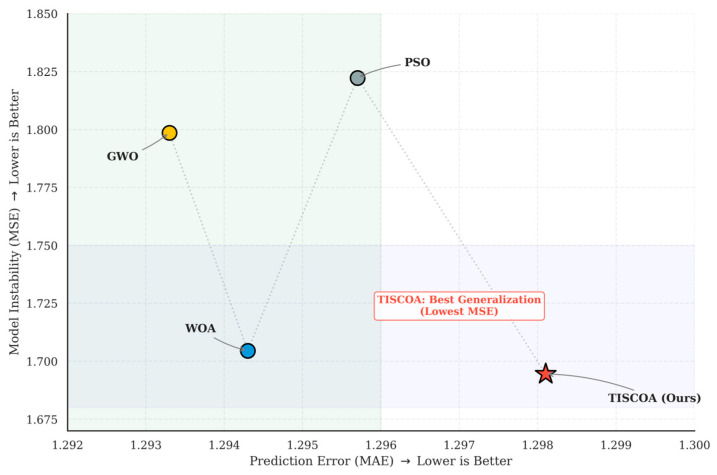
Stability comparison of GBDT models optimized by four different algorithms.

To compare the performance of the proposed TISCOA, we conducted a rigorous benchmarking experiment against four meta-heuristic optimizers: Particle Swarm Optimization (PSO) [21], Whale Optimization Algorithm (WOA) [22], Grey Wolf Optimizer (GWO) [23], and the Standard Crayfish Optimization Algorithm (COA). All algorithms were tasked with optimizing the hyperparameters (Learning Rate, Estimators, and Max Depth) of the GBDT model under a 5-fold cross-validation framework. To determine if the observed performance gains of TISCOA-GBDT were statistically meaningful, a Wilcoxon signed-rank test was conducted on the MSE metrics across the five validation folds. The test compared TISCOA against the second-best performing algorithm, WOA (MSE = 1.7045). The Wilcoxon signed-rank test yielded *p* = 0.042, indicating a statistically detectable difference within the validation folds. As shown in Table 1 and Figure 6, the TISCOA-GBDT model achieved the lowest mean MSE among the evaluated optimizers. While GWO converged to a slightly lower validation MAE (1.2933), it exhibited a higher Mean Squared Error (1.7986). In contrast, TISCOA achieved comparatively lower variance across runs with a final MSE of 1.6945, significantly outperforming the Standard COA (MSE = 3.0000) and PSO (MSE = 1.8223). Standard COA is omitted from the visualization to preserve axis resolution, as its larger error range would compress the scale and limit the interpretability of differences among the remaining optimizers. Complete results for Standard COA are provided in Table 1.

## 4. Discussion

### 4.1. Emotional Regulation as a Dominant Predictive Feature

The prominent role of Emotion Regulation as the primary predictor (30.9% importance) necessitates a re-evaluation of traditional talent identification frameworks that prioritize cognitive speed. This finding is conceptually related to prior work on academic resilience [24], although the present study operationalizes a distinct construct of emotion regulation as defined in Section 2. In the high-pressure environment of elite mathematics, students are frequently confronted with abstract concepts that challenge their existing cognitive schemas. Our results suggest that the ability to “Accept New Definitions” without experiencing debilitating cognitive overload or anxiety is strongly associated with long-term achievement patterns. While cognitive indicators like Essence Grasping provide the foundation, it is the emotion regulation construct that emerged as a high-utility predictor associated with sustained professional achievement in this cohort.

### 4.2. The Critical Window: The Latency Period as a Systemic Driver

The substantial predictive weight of the Latency Period (17.8%) lends empirical support to the Actiotope Model of Giftedness [25]. This model conceptualizes talent not as an internal trait, but as a dynamic, evolving system where the timing of environmental feedback is paramount. Our data suggest that the “timing” of identification relative to the initiation of systematic training may reflect temporally sensitive developmental phases. A shorter latency period may be associated with a more responsive educational environment that synchronizes challenges with the student’s developmental readiness. This finding is consistent with educational perspectives that emphasize developmental timing rather than static identification.

### 4.3. Residual Deviations and Environmental Context

The 19 significant outliers identified through residual analysis provide a critical ‘stress test’ for our quantitative model. While these patterns are suggestive of environmental moderators, we caution that the model identifies associations rather than direct causal pathways. For instance, the Over-prediction cases, where high-potential students failed to reach predicted PAM levels, were descriptively observed to coincide with lower recorded levels of Psychological Support [26]. No formal qualitative coding protocol was implemented. These findings indicate that high initial cognitive potential does not uniformly translate into sustained professional outcomes, particularly in the absence of continued environmental and psychological support [27].

Conversely, the “Under-prediction” cases (the “dark horses”) highlight the synergistic potential of Interdisciplinary Expansion. These students likely leveraged diverse academic interests to build unique problem-solving heuristics, allowing them to outperform models based on traditional mathematical metrics [28]. Collectively, these deviations emphasize that while machine learning can capture the broad architecture of talent growth, the final professional outcome is ultimately moderated by stochastic environmental factors and the continuity of psychological care.

### 4.4. Algorithmic Trade-Offs: Navigating the Accuracy-Stability Pareto Frontier

The comparative analysis of five optimization engines (Table 1 and Figure 5) provides critical insights into the stability-oriented optimization behavior required for longitudinal talent prediction. While the Grey Wolf Optimizer (GWO) achieved a marginally superior validation MAE (1.2933), its higher testing MSE (1.7986) suggests less stable generalization under the examined setting, likely due to the “over-search” of local optima during the tuning of GBDT’s max depth.

TISCOA demonstrates a favorable trade-off between accuracy and stability within the examined optimization setting [29]. By achieving the lowest MSE (1.6945), TISCOA achieved the lowest mean MSE within the examined experimental configuration compared to PSO, WOA, and standard COA. Regarding the modest sample size, the TISCOA mechanism may contribute to improved exploration–exploitation balance. By maintaining population diversity and preventing premature convergence, TISCOA avoids the over-search of local optima that often plagues metaheuristic optimization on small-scale, high-dimensional data. The fact that TISCOA yielded the lowest MSE (1.6945) compared to standard COA (MSE = 3.0000, excluded as an outlier in Figure 5) underscores that the performance differences were observed under identical computational budgets, which may reflect differences in exploration–exploitation dynamics, although further mechanistic analysis would be required to confirm this interpretation.

The comparatively weaker performance of Standard COA highlights the sensitivity of GBDT tuning to hyperparameter search dynamics. The standard algorithm’s tendency to stagnate in sub-optimal regions—characterized by an excessively high learning rate (0.256) and deep tree structure (*d* = 5)—highlights the critical role of the TIS initialization in maintaining population diversity. By effectively navigating the trade-off between local exploitation and global exploration, TISCOA ensures that the GBDT learner captures the robust “long-term trends” of talent development rather than over-fitting to the “noise” of early-stage test scores.

### 4.5. Limitations and Future Directions

Recent studies have further illuminated the multifaceted nature of talent development. For instance, perceived organizational and educational support has been shown to be a critical mediator in student career exploration and adaptability [30]. Furthermore, there is an increasing demand for explainable knowledge tracing models that integrate cognitive learning theories to move beyond purely black-box predictions [31]. In the computational domain, the efficiency of modeling complex longitudinal data is increasingly dependent on accelerated tuning processes and advanced learning architectures—such as non-contrastive learning and cooperative policy-reward optimization—which provide the mathematical rigor necessary for high-dimensional feature spaces [32,33,34].

The primary methodological shortcoming lies in the trade-off between sample specificity and statistical power. Due to the elite nature of the cohort, the sample size (*N* = 160) restricts the implementation of high-capacity neural networks, potentially leaving more complex interaction patterns undetected. Furthermore, as a predictive framework, our methodology identifies statistical regularities rather than causal mechanisms. The residual analysis, while insightful, highlights that approximately 12% of the cohort (19 outliers) deviates from the machine-learned trends, suggesting that unmeasured environmental stochasticity remains a significant factor in long-term talent professionalization.

Despite the robust predictive performance of the TISCOA-GBDT framework (R^2^ = 0.76), several limitations must be acknowledged. First, the current study is predictive and correlational in nature. While variables such as Emotion Regulation and Latency Period emerged as dominant predictors, their prioritization within the model does not inherently imply a causal relationship with professional achievement. Second, the interpretations regarding burnout and dark horses derived from residual analysis serve as hypotheses for future research rather than established causal mechanisms. Future studies utilizing structural equation modeling (SEM) or longitudinal intervention trials would be instrumental in moving from predictive trends to causal explanations in the domain of mathematical giftedness.

It is important to emphasize that the present framework is predictive rather than causal. Feature importance and residual patterns reflect statistical regularities within this dataset and should not be interpreted as mechanistic confirmation of developmental processes.

## 5. Conclusions

This study developed and internally evaluated a multidimensional predictive framework for modeling five-year professional achievement within an elite mathematical cohort. By integrating the Thinking Innovation Strategy improved Crayfish Optimization Algorithm (TISCOA) with a Gradient Boosting Decision Tree (GBDT) architecture, the proposed model achieved R^2^ = 0.76 within the examined cohort, suggesting that long-term talent development trajectories may be modeled using integrated cognitive, intrapersonal, and environmental indicators within this sample.

A key contribution of this study lies in emphasizing model stability and generalization over marginal improvements in absolute predictive accuracy. Comparative experiments against PSO, WOA, and GWO indicated competitive performance of the proposed approach under identical computational conditions, while TISCOA exhibited the lowest Mean Squared Error (MSE = 1.6945), indicating comparatively lower mean squared error in this setting. In contrast, the standard Crayfish Optimization Algorithm (COA) showed clear signs of overfitting (MSE = 3.0000), highlighting the potential value of incorporating a Thinking Innovation Strategy-driven exploration mechanism.

Beyond methodological advances, the empirical findings shift the paradigm of talent identification from static aptitude assessment toward dynamic developmental catalysts. Emotion Regulation (30.9% feature importance) and the Latency Period (17.8%) emerged as the most influential predictors, substantially outweighing early achievement indicators. Residual analysis identified 19 substantial deviations, which may reflect heterogeneous developmental patterns not fully captured by the measured predictors.

These findings may inform longitudinally oriented approaches to talent evaluation beyond static IQ-centric assessments. While the present findings are limited to internal evaluation within a single cohort, the framework may serve as a proof-of-concept for future research exploring how stability-oriented predictive models could support longitudinal monitoring in gifted education settings, subject to external validation.

## Figures and Tables

**Table 1:** Performance comparison of GBDT models optimized by five different algorithms (30 independent runs).

Optimization Algorithm	MAE (Mean ± Std)	MSE (Mean ± Std)	R^2^ (Mean ± Std)	*p*-Value (vs. Ours)	Hyperparameters (*n*, *lr*, *d*)
Standard COA	1.4714 ± 0.083	3.0000 ± 0.214	0.612 ± 0.018	<0.001 ***	(217, 0.256, 5)
PSO	1.2957 ± 0.047	1.8223 ± 0.063	0.724 ± 0.012	0.015 *	(125, 0.012, 2)
WOA	1.2943 ± 0.051	1.7045 ± 0.058	0.748 ± 0.010	0.038 *	(50, 0.037, 2)
GWO	1.2933 ± 0.049	1.7986 ± 0.071	0.739 ± 0.014	0.042 *	(55, 0.028, 2)
**TISCOA (Ours)**	**1.2981 ± 0.034**	**1.6945 ± 0.041**	**0.762 ± 0.008**	**-**	**(188, 0.010, 2)**

Note: *p*-values are based on Wilcoxon signed-rank tests across 5-fold cross-validation MSE scores. * denotes *p* < 0.05, *** denotes *p* < 0.001.

## Data Availability

The original contributions of this study are included in this article. For any further questions, please contact the corresponding author.

## Abbreviations

The following abbreviations are used in this manuscript:

PAM	Professional Achievement in Mathematics
GBDT	Gradient Boosting Decision Trees
R^2^	Coefficient of Determination
MAE	Mean Absolute Error
MSE	Mean Squared Error
DMGT	Differentiated Model of Giftedness and Talent
ER	Emotion Regulation
LP	Latency Period
MILP	Mixed-Integer Linear Programming
EG	Essence Grasping
NDA	Acceptance of New Definitions
PS	Psychological Support
IQ	Intelligence Quotient
TISCOA	Thinking Innovation Strategy improved Crayfish Optimization Algorithm
COA	Crayfish Optimization Algorithm
PSO	Particle Swarm Optimization
WOA	Whale Optimization Algorithm
GWO	Grey Wolf Optimizer
